# Hospital and Institutional News

**Published:** 1911-12-30

**Authors:** 


### HOSPITAL AND INSTITUTIONAL NEWS.

### SIR ERNEST CASSEL'S GIFT.

Christmas Day and the New Year, which start almost simultaneously, have received, from the hospital's point of view, a magnificent send off by Sir Ernest Cassel's gift of £50,000, which became known on Christmas Eve. This sum, which has been given in memory of the late Mrs. Maud Ashley, the daughter of the giver, is not confined to hospitals only, but includes more than one convalescent home and nursing institution. Its perhaps most interesting provision is that by which £31,500 goes to King Edward's Hospital Fund for London, on the understanding that it be divided among the institutions which receive grants from the Fund this year in the same proportion as that in which the Fund's grants are divided. This will increase the distribution of the Fund by one-fifth—from £157,500 to £189,000. The remainder has been allocated among various London hospitals, subject to certain conditions, except £7,000 which is divided between the Romsey Nursing Home, Romsey, Hampshire; the Royal Hants County Hospital, Romsey Road, Winchester, Hampshire; the Victorian Hospital, Blackpool, Lancashire; the Fleetwood Cottage Hospital, Fleetwood, Lancashire; the Lytham Cottage Hospital, Lytham, Lancashire; the Royal National Hospital for Consumption and Diseases of the Chest, Ventnor; the Royal Sea-Bathing Hospital, Margate. Everyone will be glad to see the hospital outlook thus suddenly brightened, and to know that the hands of the King's Fund have been strengthened at the outset of the New Year.

### THE BEIT RESEARCH FELLOWSHIPS.

A fresh list of gentlemen elected by the Trustees of Mr. Beit's foundation, with the subjects proposed for investigation, has been published. The Fellowships, it will be remembered, are tenable usually for three years, and are of the annual value of £250. It is noteworthy that of the ten new Fellows, one is a German, one a Canadian, and one an Australian with a Hebrew name, while a fourth proposes to carry out his researches in Munich. Three, again, are not members of the medical profession, but are to be engaged in chemical or biological researches which have an important bearing on medical problems. The trustees have evidently interpreted their functions with a catholic and cosmopolitan outlook. Dr. Bayou proposes to do bacteriological work especially connected with the problems of leprosy, and will establish himself in a leper camp or colony for that purpose. Mr. Cooper is to work at the ætiology of beri-beri. Miss Fraser will inquire into the value of the complement fixation test in tuberculosis. Dr. Graham will devote himself to the retention of nitrogen in kidney disease. Dr. Gunn has four lines of inquiry, mainly pharmacological: one of these concerns hormaline as a possible substitute for, or adjuvant to, ergot. Dr. Harvey will examine the effects of urea, caffeine, and other substances in producing pathological conditions of the kidney. Dr. Jona takes the important subject of toxæmias attendant upon pregnancy and childbirth. Mr. Norris will investigate the formation and metabolism of glycogen and its bearing upon diabetes. Mr. O'Donoghue's research is to be concerned with the œstrus cycle in relation to the functional activity of the mammary glands, and the whole physiology of the mammary apparatus. Dr. Twort will examine into Johne's disease of cattle and its connection with human, bovine, and avian tuberculosis. Four out of the ten Fellows will work in the Nester Institute, Chelsea, and two at University College.

### THE NATIONAL MEDICAL UNION.

The National Medical Union held an important meeting of its Provisional Executive Committee on the 22nd inst. The official report intimates that the subscription for membership has been fixed at half a guinea. The Union extends cordial support to every federation of associations and unions of medical men formed to create a fund to provide against financial loss resulting from the National Insurance Act. The Chairman of the Union is Mr. G. A. Wright, F.R.C.S., deputy-chairman, Dr. William Coates, C.B., the treasurer Dr. T. Arthur Helme, and the secretary Dr. T. Wheeler Hart—all of Manchester. The following embrace the main features of the official programme of this new Union: (1) All members of the National Medical Union should promise to oppose the medical service of the National Insurance Act, 1911, by declining to go on any panel or committee, and by refusing to undertake any of the duties which the Act proposes to assign to them. (2) To strengthen the hands of the members of the British Medical Association and all other societies engaged in safeguarding the interests of the medical profession. (3) Each division or group of members shall have the right on all vital points of policy to claim a referendum of the whole Union.

### "THE SCANDALS AT GUY'S HOSPITAL."

The old proverb " Its an ill wind that blows nobody any good " is nowhere better illustrated than in the history of Guy's Hospital. During the last few years the repetition of amazing complaints against the administration of this South London charity has attracted public attention to the hospital, and has been the means of gaining Guy's more than one good friend who has testified his or her sympathy and confidence in the work that the hospital is carrying on, in a manner that is thoroughly practical. Lately we have had several grievances against the hospital ventilated in the Press, and the public has also had an opportunity of reading the calm and reasoned defence put forward by the medical superintendent of the hospital. Those who know the facts will have little difficulty in deciding between accuser and defendant in this case. Unfortunately however the public does not know the facts, and the more publicity is given to the reasons which prompt subscribers to remain loyal to the institution the better will the management be able to deal with the mischievous and ignorant criticism which does so much harm to this hospital and other charities.

### A SUBSCRIBER'S APPRECIATION OF THE WORK THAT IS DONE AT GUY'S.

We venture to think that the governors would do well to make public the letter which has lately been received from one who styles himself "An Old Guy's Man." This letter is written as the direct result of the ventilation of these so-called grievances against Guy's in the local Press, and we make no apology for quoting part of it here, since it shows very clearly the reasoned appreciation which those of us who are acquainted with the work that is daily carried on at Guy's must feel for the governors and those in charge of the hospital. "A friend of mine," the writer remarks, "desirous of remaining anonymous, wishes in some way to mark his recognition of the great work that is being carried on by Guy's Hospital, and of the efficient and unostentatious way in which it does that work. In common with many others he has observed with regret the attacks which from time to time, and especially lately, have been made upon the hospital authorities in certain organs of the Press. He is much impressed by the adequacy and restraint of the defence as detailed in the Superintendent's letter" (published in our issue of December 2) "and he wishes as heartily as all friends of Guy's must, that as wide a circulation as possible could be assured to that defence as has been vouchsafed to the attack. . . . He feels that the public do not, and cannot, appreciate properly the enormous anxiety and responsibility which attach to the chief administrative posts in a great hospital, an anxiety which is accentuated in these days of democracy and cheap newspapers, and he wishes to give some proof of the admiration he feels for the magnificent service rendered by the Medical Superintendent during the past 20 years. Accordingly he offers a sum of £1,000 to the hospital, the interest of which sum is to be "expended for the benefit of the men trained or in training at Guy's in some way that the Superintendent may think desirable, provided only that the choice shall lie with Sir Cooper himself, and that he shall devote the money to some object which he shall judge most useful." This is a model of what such a mark of appreciation should be, and we congratulate both the governors of Guy's Hospital and Sir Cooper Perry, the Superintendent, on the almost unique compliment which they have received from this anonymous friend.

### SIR WILLIAM BULL'S HOSPITAL RECORD.

There are some interesting facts to note in connection with the election of Sir William Bull, M.P., to the Board of Management of the West London Hospital. Sir William is already known in the hospital world, for he has been for twenty years a member, and, during the past year, chairman, of the Committee of Management of the Seaside Convalescent Hospital, Seaford, Sussex. He is also a trustee under the will of the late Mr. Richard Smith, known to the public in relation to Hovis bread, and, to hospital treasurers as a man who left a large reversionary legacy to the Metropolitan Ear, Nose and Throat Hospital. Sir William is also a member of the council and trustee of the Royal Humane Society. It will be seen from this that a practical experience of routine work has proved the basis of Sir William Bell's institutional record, and as such he is entitled to the respect of the hospital world.

### QUEEN'S HOSPITAL FOR CHILDREN.

Like one or two other institutions just now the Queen's Hospital for Children, Hackney Road, Bethnal Green, is racing against time in order to secure a conditional promise of £500. Mr. T. Glenton Kerr, the secretary, is waiting for cheques, and it requires very little imagination to remember that the North East End is as much poverty stricken, and not so much "written up" as the conventional slum of Whitechapel.

### COUNTY COUNCIL'S HOSPITAL CONTRIBUTIONS.

The London County Council has decided, with the powers obtained by an Act of Parliament, to allocate not more than £500 a year for contributions to any hospital, convalescent home, dispensaries, or other similar institutions in which any employee of the Council may receive treatment. In the remaining three months of the present financial year it is proposed to allocate £100, of which roughly £15 will be contributed to hospitals and £85 to convalescent homes. A factor taken into account will be whether employees are or are not entitled to medical attendance at the expense of the Council. Major Levita, speaking as the manager of a hospital, welcomed any contributions they could receive, but said that the amount proposed was not large enough to entitle the Council to preferential treatment. His hospital expended more than the total amount the Council proposed to subscribe on tuberculous employees of the Council alone. Major Levita suggested that the Council should make arrangements with the hospitals to pay a certain amount and have the first claim to certain beds. On behalf of the General Purposes Committee, which is to carry out the distribution, Mr. H. J. Greenwood agreed that this was only the first step.

### A ROYAL COMMISSION ON QUACKERY.

The scandalous way in which the entire British Press—with two or three honourable exceptions—boycotted the British Medical Association's publication "Secret Remedies" in 1909 is a standing record of the complete power of the nostrum-vendors and their advertisements and of the ignoble cowardice of newspaper proprietors. The special "quackery" number of the *British Medical Journal* some six months ago met with a similar fate. But signs are not wanting that the leaven is beginning to work, and in private practice it is now by no means unknown for patients to discuss "Secret Remedies"

with their doctors; and one lady recently informed her medical man that she found it one of the most amusing books she had ever read. Encouraging as these signs of light in darkness are, there is need for many more torchbearers before truth can be properly revealed to the multitude. Of those who have kept the flame alight hitherto, honourable mention is especially due to Dr. Henry Sewill, whose addresses and contributions to *Vanity Fair* have long been means of enlightenment both to the public and the medical profession.

### DR. SEWILL'S AGITATION.

It is with great pleasure that we notice that Dr. Sewill is still agitating for a Royal Commission as the best way to unsettle the conspiracy of silence of the Press as a whole; and there can be little doubt that such a Commission offers the best prospect of making headway against the gigantic evil of quackery. In a pamphlet recently issued are reprinted several of Dr. Sewill's published articles ("Quackery," P. S. King & Son, price 6d.), designed for lay readers. Dr. Sewill appeals for the support of the lay and medical experts who appreciate the magnitude of the evils which he seeks to combat. We trust the pamphlet will have a large circulation among those whom it is designed to benefit, and that the suggested Royal Commission may soon come to be an accomplished fact.

### BRADFORD'S ANTI-PHTHISIS SCHEME.

Dr. Arnold Evans, Medical Officer of Health to Bradford, is urging the local Health Committee to found a tuberculosis dispensary on the Edinburgh system. This system was carefully summarised in The Hospital of November 25 (page 194), and our readers will therefore appreciate the distinction which Dr. Evans draws between a "dispensary" and what he prefers to call a "tuberculosis clinic." As we pointed out, the essentials in fighting phthisis in towns are a sorting centre, and correlation of clinic and sanatorium with the patient's homes. Such dispensaries, to revert to their popular title, should form the clearing houses of the various districts and should be the depository and register of invaluable statistics. Dr. Evans has made out a very strong case, and his report should be memorable for once more driving home that "the Edinburgh system has been adopted by the Local Government Board of Scotland as a national scheme for that country; it has been described by Dr. Herman Biggs, Medical Officer of Health for New York, as a perfect organisation." Indeed it is the one on which he has based the whole of his anti-tuberculous measures for that city. Let us hope that Bradford will support her Medical Officer as strongly as he certainly deserves.

